# Cytoplasmic dynein regulates the subcellular localization of sphingosine kinase 2 to elicit tumor-suppressive functions in glioblastoma

**DOI:** 10.1038/s41388-018-0504-9

**Published:** 2018-09-24

**Authors:** Heidi A. Neubauer, Melinda N. Tea, Julia R. Zebol, Briony L. Gliddon, Cassandra Stefanidis, Paul A. B. Moretti, Melissa R. Pitman, Maurizio Costabile, Jasreen Kular, Brett W. Stringer, Bryan W. Day, Michael S. Samuel, Claudine S. Bonder, Jason A. Powell, Stuart M. Pitson

**Affiliations:** 1 Centre for Cancer Biology, University of South Australia and SA Pathology, Adelaide, SA Australia; 2 School of Biological Sciences, University of Adelaide, Adelaide, SA Australia; 3 School of Pharmacy and Medical Sciences, University of South Australia, Adelaide, SA Australia; 4 QIMR-Berghofer Medical Research Institute, Brisbane, QLD Australia; 5 Adelaide Medical School, University of Adelaide, Adelaide, SA Australia

**Keywords:** CNS cancer, Cell signalling

## Abstract

While the two mammalian sphingosine kinases, SK1 and SK2, both catalyze the generation of pro-survival sphingosine 1-phosphate (S1P), their roles vary dependent on their different subcellular localization. SK1 is generally found in the cytoplasm or at the plasma membrane where it can promote cell proliferation and survival. SK2 can be present at the plasma membrane where it appears to have a similar function to SK1, but can also be localized to the nucleus, endoplasmic reticulum or mitochondria where it mediates cell death. Although SK2 has been implicated in cancer initiation and progression, the mechanisms regulating SK2 subcellular localization are undefined. Here, we report that SK2 interacts with the intermediate chain subunits of the retrograde-directed transport motor complex, cytoplasmic dynein 1 (DYNC1I1 and -2), and we show that this interaction, particularly with DYNC1I1, facilitates the transport of SK2 away from the plasma membrane. DYNC1I1 is dramatically downregulated in patient samples of glioblastoma (GBM), where lower expression of DYNC1I1 correlates with poorer patient survival. Notably, low DYNC1I1 expression in GBM cells coincided with more SK2 localized to the plasma membrane, where it has been recently implicated in oncogenesis. Re-expression of DYNC1I1 reduced plasma membrane-localized SK2 and extracellular S1P formation, and decreased GBM tumor growth and tumor-associated angiogenesis in vivo. Consistent with this, chemical inhibition of SK2 reduced the viability of patient-derived GBM cells in vitro and decreased GBM tumor growth in vivo. Thus, these findings demonstrate a tumor-suppressive function of DYNC1I1, and uncover new mechanistic insights into SK2 regulation which may have implications in targeting this enzyme as a therapeutic strategy in GBM.

## Introduction

Sphingosine 1-phosphate (S1P) is an important signaling lipid that regulates many cellular processes, including cell survival, proliferation, apoptosis, migration and differentiation [1]. Cellular S1P is generated from sphingosine by the sphingosine kinases (SKs), SK1 and SK2. Many signaling pathways activated by S1P promote cell survival and proliferation, whereas sphingosine, and its precursor ceramide, are both pro-apoptotic molecules [2]. Interestingly, despite both enzymes catalyzing the formation of S1P, SK1 and SK2 have both overlapping and divergent functions within the cell [1, 3], with these differing functions dictated by their differential subcellular localization [4].

SK1 is largely cytoplasmic, but we have previously shown that phosphorylation and activation promotes SK1 translocation to the plasma membrane [5] where it can facilitate pro-survival, pro-proliferative signaling [6–8]. As such, targeting SK1 has demonstrated anti-tumor effects [9]. Similarly, we recently showed that SK2 can mediate tumorigenesis, and it too was found localized to the plasma membrane to increase S1P levels in this setting [9]. However, the roles of SK2 are complex and, unlike SK1, SK2 can also promote cell cycle arrest and cell death under certain conditions, where these functions appear to require changes to SK2 subcellular localization. Specifically, SK2 localization to the nucleus and internal organelles can confer pro-apoptotic, anti-proliferative functions, whereas plasma membrane localization drives pro-proliferative and oncogenic signaling [3]. However, the mechanisms regulating SK2 translocation between various cellular compartments in order to effect changes in its functions are currently unexplored.

In this study, we identified cytoplasmic dynein 1 intermediate chains 1 and 2 (DYNC1I1 and DYNC1I2) as SK2-interacting proteins. We demonstrate that SK2 interacts with the cytoplasmic dynein complex in cells and, consistent with the retrograde transport function of dynein, interaction with DYNC1I1-containing dynein complexes appears to facilitate transport of SK2 away from the cell periphery. Furthermore, we report a dramatic downregulation of DYNC1I1 in glioblastoma (GBM), which correlates with poorer patient survival, and demonstrate that lower DYNC1I1 expression in GBM cells coincides with more SK2 localized to the plasma membrane. Notably, re-expression of DYNC1I1 in GBM cells reduced plasma membrane-localized SK2 and extracellular S1P formation and, strikingly, decreased tumor growth and tumor-associated angiogenesis in vivo. Consistently, chemical inhibition of SK2 showed efficacy against human GBM patient-derived cells in vitro and decreased GBM xenograft tumor growth in vivo. Together, our findings indicate a novel tumor-suppressive function of DYNC1I1 in GBM via dynein-mediated regulation of SK2.

## Results

### Dynein intermediate chains are SK2-interacting proteins

**Fig. 1:**
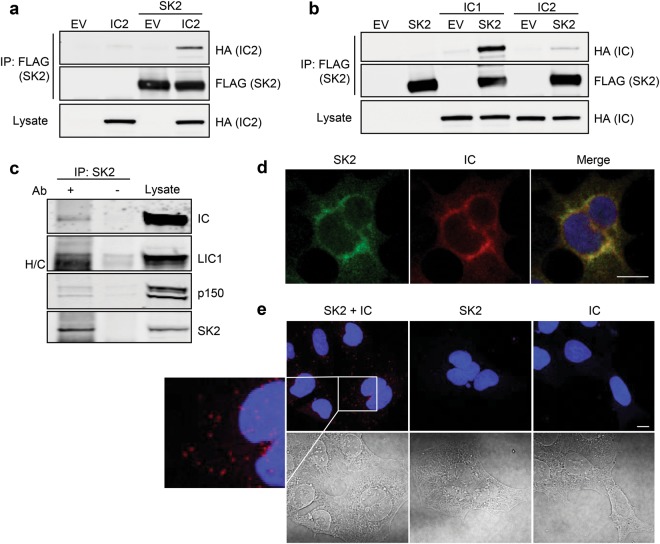
SK2 interacts with dynein IC. **a** HEK293 cells were transfected with empty vector (EV) or a vector encoding HA-tagged IC2, either individually or in combination with a vector encoding FLAG-tagged SK2. SK2 was immunoprecipitated from cell lysates with anti-FLAG antibodies, and co-immunoprecipitated IC2 was detected by immunoblotting with anti-HA antibodies. Expression levels of IC2 in the lysates were confirmed by immunoblotting with anti-HA antibodies (Lysate). Immunoprecipitates were also probed with anti-FLAG antibodies to confirm the presence of SK2. Blots shown are representative of at least five independent experiments. **b** HEK293 cells were transfected with empty vector (EV) or a vector encoding FLAG-tagged SK2, either individually or in combination with a vector encoding HA-tagged IC1 or HA-tagged IC2. Lysates were pre-cleared with Protein G µbeads. Co-immunoprecipitation and immunoblotting analyses were then performed as described in (**a**). Blots shown are representative of three independent experiments. **c** SK2 was immunoprecipitated from murine whole brain lysate using anti-SK2 antibodies. Co-immunoprecipitated dynein intermediate chains (IC), light intermediate chain 1 (LIC1) and dynactin p150 were detected by immunoblotting with anti-IC, anti-LIC1 and anti-dynactin p150 antibodies, respectively. Expression levels of these proteins in the mouse brain lysate were confirmed by immunoblot analyses with their respective antibodies (Lysate). Lysates and immunoprecipitates were also probed with anti-SK2 antibodies to confirm expression and immunoprecipitation of SK2. H/C designates the heavy chain IgG band. Blots shown are representative of three independent experiments. **d** Immunofluorescence staining and confocal microscopy demonstrating co-localization of SK2 and dynein IC in HEK293 cells. SK2 (green) was detected using anti-SK2 antibodies and dynein IC (red) was detected using anti-IC antibodies. Nuclei were highlighted using DAPI (blue). Images are representative of at least 100 cells, from three independent experiments. Scale bar = 10 μm. **e** Immunofluorescence analysis and confocal microscopy demonstrating direct interactions between SK2 and dynein IC, using the Duolink® in situ PLA system with anti-SK2 (1:300; ECM Biosciences) and anti-IC antibodies (1:300) in HEK293 cells (top panels). Each red dot indicates a single direct interaction. Nuclei were highlighted using DAPI (blue). Differential interference contrast images are also shown (bottom panels). Images are representative of at least 100 cells, from three independent experiments. Scale bar = 10 μm

To better understand the mechanisms of SK2 regulation, a yeast two-hybrid screen was performed to identify novel SK2-interacting proteins. One candidate protein was identified as cytoplasmic dynein 1 intermediate chain 2 (DYNC1I2; hereafter referred to as IC2), as represented by the isolation of a partial complementary DNA (cDNA) encoding the *c*-terminal 173 amino acids of this 67 kDa protein (residues 440–612). An association between full-length IC2 and SK2 was verified by co-immunoprecipitation in mammalian cells co-expressing the two proteins (Fig. 1a). Two mammalian cytoplasmic dynein intermediate chain isoforms exist, the ubiquitously expressed IC2 identified in the yeast two-hybrid screen and IC1 (DYNC1I1) which is expressed most abundantly in the brain [10]. Interestingly, compared to IC2, the interaction between SK2 and IC1 appeared stronger (Fig. 1b), potentially suggesting an important isoform-specific role for IC1 in interacting with SK2.

Next, to confirm interaction of the endogenous proteins, SK2 was immunoprecipitated from murine brain lysates, which demonstrated a robust co-immunoprecipitation of endogenous dynein intermediate chains (ICs; Fig. 1c). Given that the ICs are found physiologically as dimeric subunits of the dynein complex, the presence of other dynein components within the SK2 immunocomplexes was examined. Indeed, an association of SK2 with the dynein light intermediate chain 1 (LIC1) subunit was observed, and also with the p150 subunit from the dynactin complex (Fig. 1c), which is a regulatory complex that plays important roles in facilitating almost all dynein functions [11].

Immunofluorescence analysis of endogenous SK2 and dynein ICs was then performed to examine their localization. Consistent with their physiological interaction, both proteins showed considerable subcellular co-localization in HEK293 cells, mainly at cytoplasmic and peri-nuclear regions (Fig. 1d). Further confirmation of the physiological interaction of endogenous SK2 and dynein ICs was obtained by in situ proximity ligation assays (PLAs), which again demonstrated a clear SK2–IC interaction mainly at cytoplasmic and peri-nuclear regions (Fig. 1e). Appropriate single antibody controls confirmed that detection of both proteins together was required to produce PLA signals (Fig. 1e). Taken together, these data demonstrate that SK2 interacts with dynein ICs as a part of the physiological dynein complex in cells.

### SK2 subcellular localization is regulated by dynein

**Fig. 2:**
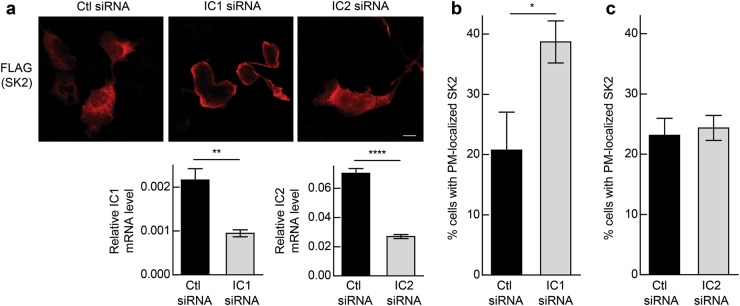
Dynein regulates the subcellular localization of SK2. **a** Immunofluorescence analysis and confocal microscopy of HEK293 cells transfected with a vector encoding FLAG-tagged SK2 and either negative control (Ctl) siRNA, IC1 siRNA or IC2 siRNA for 48 h. SK2 (red) was detected using anti-FLAG antibodies. Images are representative of at least 200 cells observed from three independent experiments. Scale bar = 10 µm. IC1 and IC2 knockdown was assessed by qRT-PCR with levels of IC1 and IC2 mRNA normalized to GAPDH mRNA (**p < 0.01, ****p < 0.0001; Student's unpaired two-tailed *t*-test). Cells with knockdown of IC1 (**b**) or IC2 (**c**) were scored based on the presence of plasma membrane (PM)-localized FLAG-tagged SK2. A minimum of 200 cells were scored per treatment. Data were graphed as mean (±SD) of triplicate wells from a single experiment, representative of three independent experiments (*p < 0.05; Student's unpaired two-tailed *t*-test)

The interaction and subcellular localization of SK2–IC complexes is consistent with the role of dynein in transporting cargo in a retrograde direction along microtubules, which eventually accumulate at the microtubule-organizing center (MTOC) located adjacent to the nucleus [12, 13]. Given that SK2 co-localized with dynein ICs mainly at peri-nuclear regions of the cell, the involvement of dynein in the retrograde-directed transport of SK2 was examined. To do this, small interfering RNA (siRNA)-mediated IC1 and IC2 knockdown was performed in HEK293 cells which express both IC proteins, and ectopic expression of SK2 was employed to better visualize its subcellular localization. Consistent with the localization of endogenous SK2 in these cells, ectopically expressed SK2 had a predominantly peri-nuclear localization (Fig. 2a). Upon RNA interference (RNAi)-mediated knockdown of IC1 however, this changed to strong plasma membrane localization of SK2 (Fig. 2a, b). Notably, however, no effect on the subcellular localization of SK2 was observed with IC2 knockdown (Fig. 2a–c). Therefore, these data are consistent with the notion that SK2 is transported by IC1-containing dynein complexes in a retrograde direction away from the plasma membrane.

### Dynein IC1 is downregulated in glioblastoma and correlates with poor patient survival

At the plasma membrane, S1P produced by the SKs can be exported from the cell where it can act on a family of five S1P receptors to promote cell survival and proliferation, as well as contribute to oncogenic signaling pathways [1]. This role is well established for SK1 [6], and we have recently reported a role for SK2 in mediating oncogenesis which coincided with increased localization of SK2 at the plasma membrane and increased extracellular S1P formation [9]. Therefore, the increase in plasma membrane-localized SK2 upon dynein IC knockdown raised the question of whether downregulation of the dynein ICs could contribute to an oncogenic phenotype.

**Fig. 3:**
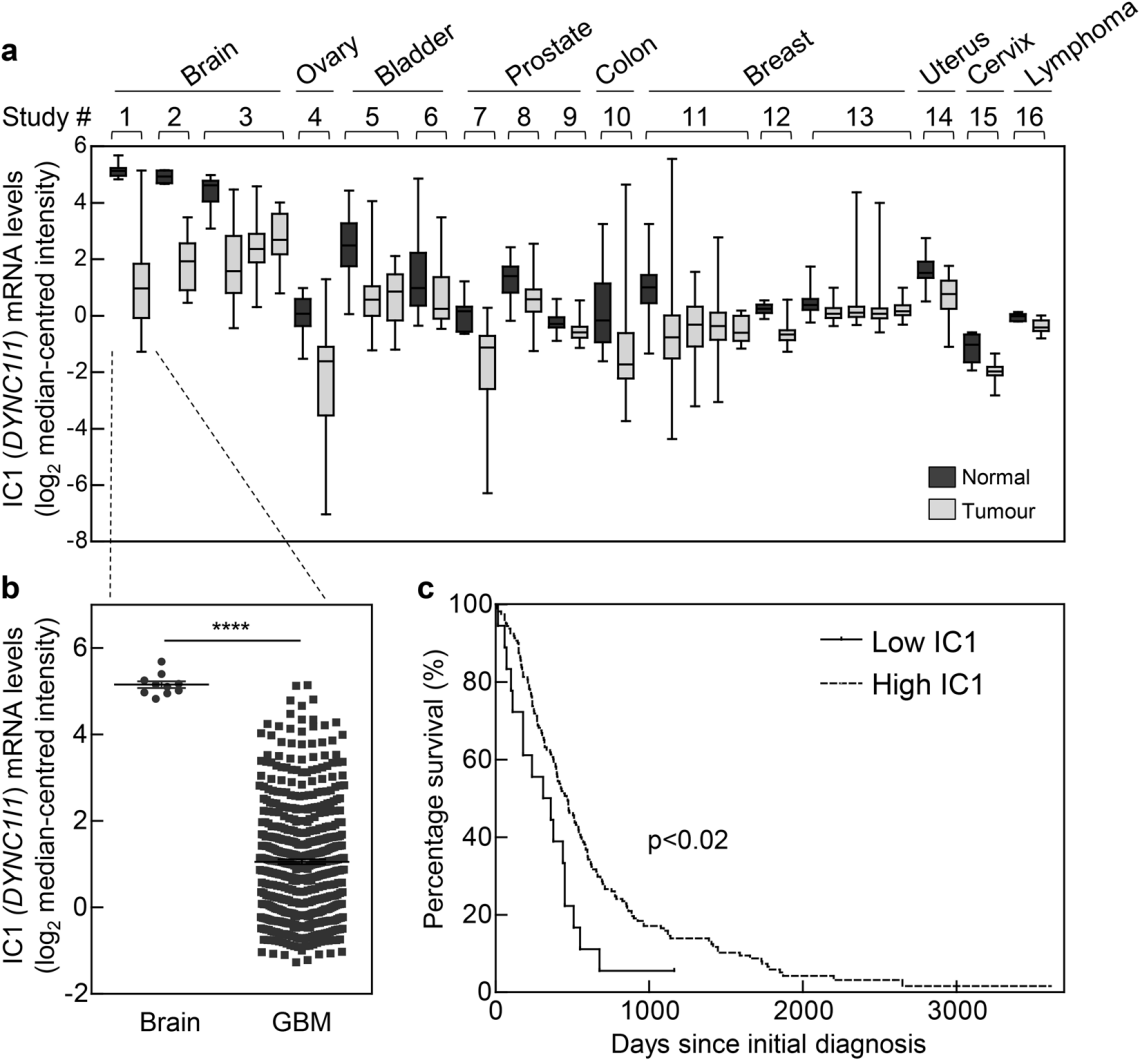
Dynein IC1 is downregulated in GBM which correlates with poorer patient survival. **a** Box plots showing human cancers with significant (*p* < 1 × 10^–4^) downregulation of IC1 (*DYNC1I1*) mRNA. Data were extracted from the Oncomine database, from the following studies (left to right): 1, TCGA Brain (glioblastoma); 2, Murat Brain (glioblastoma); 3, Sun Brain (glioblastoma, oligodendroglioma, anaplastic astrocytoma); 4, Yoshihara Ovarian (ovarian serous adenocarcinoma); 5, Sanchez-Carbayo Bladder 2 (infiltrating bladder urothelial carcinoma, superficial bladder cancer); 6, Lee Bladder (superficial bladder cancer); 7, Welsh Prostate (prostate carcinoma); 8, Lapointe Prostate (prostate carcinoma); 9, Taylor Prostate 3 (prostate carcinoma); 10, TCGA Colorectal (colon adenocarcinoma); 11, TCGA Breast (invasive ductal breast carcinoma, invasive lobular breast carcinoma, invasive breast carcinoma, mixed lobular and ductal breast carcinoma); 12, Finak Breast (invasive breast carcinoma stroma); 13, Curtis Breast (mucinous breast carcinoma, invasive lobular breast carcinoma, invasive ductal breast carcinoma, tubular breast carcinoma); 14, Crabtree Uterus (uterine corpus leiomyoma); 15, Pyeon Multi-cancer (cervical cancer); 16, Compagno Lymphoma (activated B-cell-like diffuse large B-cell lymphoma). **b** TCGA brain dataset showing a mean 17-fold downregulation of IC1 mRNA levels in human glioblastoma (GBM) tissue, compared with levels in normal human brain tissue. Statistical significance (****p < 0.0001) was determined using Student's unpaired two-tailed *t*-test. **c** IC1 mRNA expression levels and survival data from human GBM patients were obtained from the REMBRANDT dataset, and Kaplan–Meier survival curves were plotted for patients with tumors having the lowest 10% (low IC1) and highest 90% (high IC1) of IC1 expression levels. Statistical significance was determined using a two-sided log-rank test

To explore this, a broad gene expression analysis of both IC1 (*DYNC1I1*) and IC2 (*DYNC1I2*) was performed on a panel of different human cancers, using public gene expression datasets available from the Oncomine database [14]. Strikingly, IC1 was found to be significantly downregulated, compared to corresponding normal tissues, in a vast range of different cancers, including brain, ovarian, bladder, prostate, colon, breast, uterine, cervical and lymphoma (Fig. 3a). Importantly, IC2 gene expression levels in these cancer datasets were generally unaltered, compared to corresponding normal tissues (Supplementary Figure S1), demonstrating that there is no substantial compensation for IC1 loss across these cancer types. As IC1 expression is highest in brain tissues [10], it was particularly notable that a dramatic 17-fold downregulation in IC1 expression occurred in GBM patient samples compared to normal brain tissue (Fig. 3a, b). Interestingly, this was not specific to a particular GBM subtype, as IC1 expression was similarly downregulated in all three molecular subtypes of GBM [15, 16] (Supplementary Figure S2A), whereas again, IC2 expression was unchanged across all (Supplementary Figure S2B). Kaplan–Meier survival analysis of patients with GBM also demonstrated that low expression of IC1 correlated with poorer overall survival compared to patients with higher IC1 expression (Fig. 3c). Together, these analyses suggest that IC1 may play a tumor-suppressive role in cancer, and particularly in GBM.

### IC1 expression levels regulate plasma membrane localization of SK2

**Fig. 4:**
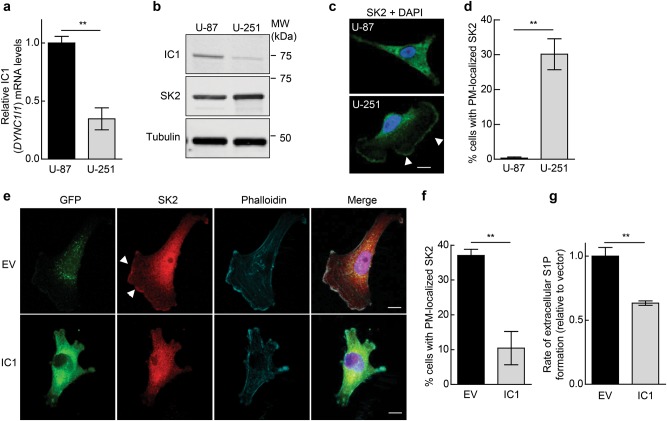
IC1 expression levels regulate plasma membrane localization of SK2. **a** Relative IC1 (*DYNC1I1*) mRNA expression levels in U-87 and U-251 human GBM cell lines. Gene expression levels were determined by qRT-PCR (comparative quantification, normalized to GAPDH). Graphs are plotted as mean (±SEM) of data from three independent experiments (**p < 0.01; Student’s unpaired two-tailed *t*-test). **b** Immunoblot analyses of IC1 and SK2 protein expression in U-87 and U-251 cell lines, using anti-IC1 and anti-SK2 (Proteintech) antibodies. Immunoblotting for α-tubulin was performed as a loading control. Blots are representative of three independent experiments. **c** Immunofluorescence staining and confocal microscopy showing endogenous SK2 localization in U-87 and U-251 GBM cells. SK2 (green) was detected using anti-SK2 antibodies (1:300; ECM Biosciences), and nuclei were stained with DAPI (blue). Arrows denote distinct plasma membrane localization of SK2. Scale bar = 10 μm. **d** Cells described above were visualized by confocal microscopy and scored based on the presence or absence of distinct plasma membrane (PM)-localized SK2 staining. A minimum of 200 cells were scored per cell type. Data were graphed as mean (±SEM) from three independent experiments (**p < 0.01; Student's unpaired two-tailed *t*-test). **e** Localization of endogenous SK2 in U-251 cells stably expressing GFP alone (empty vector; EV) or IC1-GFP was examined by confocal microscopy. IC1-GFP or GFP were visualized via their GFP tag (green) and SK2 via immunofluorescence of the endogenous protein using anti-SK2 antibodies (red; 1:300; ECM Biosciences). F-actin was stained using Phalloidin (cyan), and nuclei were highlighted using DAPI (blue). Arrows denote distinct plasma membrane localization of SK2. Images are representative of more than 100 cells, from 3 independent experiments. Scale bar = 10 μm. **f** Cells described above were visualized by confocal microscopy and scored based on the presence or absence of distinct plasma membrane (PM)-localized SK2 staining. A minimum of 200 cells were scored per cell line. Data were graphed as mean (±SEM) from three independent experiments (**p < 0.01; Student's unpaired two-tailed *t*-test). **g** The rate of extracellular S1P formation was determined from intact U-251 cells stably expressing GFP (EV) or IC1-GFP. Analyses were performed in quadruplicate and data are graphed as mean ± SD, normalized to cell number (**p < 0.01; Student's unpaired two-tailed *t*-test)

To examine the mechanisms behind the potential role of IC1 as a tumor suppressor in GBM, the expression of IC1 was initially examined in two commonly used GBM cell lines, U-87 and U-251. Notably, U-251 cells had significantly lower levels of IC1 messenger RNA (mRNA; Fig. 4a) and protein (Fig. 4b) compared with U-87 cells. Since we observed SK2 localized to the plasma membrane with IC1 knockdown (Fig. 2), we reasoned that the low levels of IC1 observed in the U-251 cells may result in increased and/or prolonged localization of SK2 at the plasma membrane. Indeed, examining the localization of endogenous SK2 by immunofluorescence staining demonstrated prominent plasma membrane localization of SK2 in U-251 cells, but not in U-87 cells (Fig. 4c, d), consistent with higher levels of IC1 in U-87 cells (Fig. 4a–d) and further suggesting a role for IC1 in regulating SK2 subcellular localization.

Next, we examined whether re-expression of IC1 in U-251 cells was sufficient to reduce SK2 localization at the plasma membrane. Taking an approach previously reported by others [17, 18], we generated U-251 cells stably expressing IC1-green fluorescent protein (GFP) at low levels, similar to endogenous IC, as confirmed by immunoblot (Supplementary Figure S3A). Importantly, exogenous IC1-GFP displayed peri-nuclear localization (Supplementary Figure S3B), formed interactions with endogenous dynein IC and LIC1 subunits (Supplementary Figure S3C) and exhibited high-level co-localization with LIC1 in cells (Supplementary Figure S3D), confirming that the fusion protein was incorporating into endogenous dynein complexes. Thus, we examined the localization of SK2 in these cells. Whereas GFP control cells had prominent plasma membrane localization of SK2, this was significantly decreased in cells overexpressing IC1-GFP (Fig. 4e, f). Concomitant with a loss of SK2 at the plasma membrane, there was also a significant reduction in the rate of extracellular S1P formation in the U-251 cells expressing IC1-GFP compared with control cells (Fig. 4g). Therefore, collectively these data demonstrate that re-expression of IC1 in U-251 GBM cells can reinstate dynein-mediated translocation of SK2 away from the plasma membrane.

### Re-expression of IC1 in U-251 GBM cells reduces neoplastic growth in vitro and in vivo

**Fig. 5:**
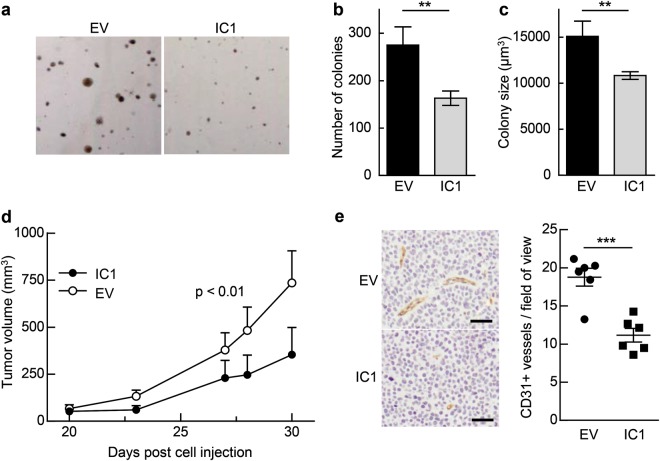
IC1 re-expression in U-251 cells reduces neoplastic growth in vitro and in vivo. **a** Anchorage-independent growth of U-251 cells stably expressing GFP alone (empty vector; EV) or IC1-GFP was evaluated using colony formation assays in soft agar. Representative images of colonies from each group are shown. Average number of colonies (**b**) and colony size (**c**) were quantified and are graphed as mean (±SD) from quadruplicate wells of one experiment, representative of three independent experiments (**p < 0.01; Student's unpaired two-tailed *t*-test). **d** NOD/SCID mice were subcutaneously engrafted with U-251 cells stably expressing IC1-GFP, or GFP alone (EV), and caliper measurements of palpable tumors were taken over 30 days. Mean tumor volumes are shown (±SEM; *n* *=* 6–7 per group). Statistical analysis on all data points was performed by two-way ANOVA. **e** Tumors derived from U-251 cells stably expressing IC1-GFP or GFP (EV) were sectioned, and CD31-positive vessels were examined by immunohistochemical staining with anti-CD31 antibodies. Representative images for each group are shown. The number of CD31-positive vessels were quantified and averaged from at least four random fields of view per tumor, at ×20 magnification, and are graphed with means (*n* *=* 6 per group; ***p < 0.001; Student's unpaired two-tailed *t*-test). All scale bars = 100 μm

As plasma membrane-localized SK can promote oncogenesis [6], we hypothesized that a loss of SK2 from the plasma membrane, and a consequent decrease in the rate of extracellular S1P formation, resulting from re-expression of IC1 may reduce the oncogenic potential of these GBM cells. Indeed, the U-251 cells re-expressing IC1 had significantly attenuated anchorage-independent growth compared to control cells, as assessed by colony formation assays in soft agar which showed a clear IC1-induced decrease in both colony number and size (Fig. 5a–c).

To determine if this reduced oncogenic potential in vitro corresponded to a decrease in tumor growth in vivo, U-251 cells re-expressing IC1 or control U-251 cells were subcutaneously engrafted into the flanks of NOD/SCID (nonobese diabetic/severe combined immunodeficiency) mice, and tumor growth measured over time. Re-expression of IC1 decreased GBM tumor growth by approximately 50% compared with tumors from control U-251 cells (Fig. 5d). The presence of re-expressed IC1 in the excised tumors was confirmed by immunoblot analyses (Supplementary Figure S4). Reduced tumor growth can be a consequence of decreased infiltrating blood vessels, limiting nutrients and oxygen available to the tumor. Since extracellular S1P plays an important role in mediating angiogenesis and blood vessel infiltration into tumor tissue [19], and given that we observed reduced extracellular S1P upon stable overexpression of IC1 in vitro, we assessed tumor vascularization by CD31 staining of tumor sections. Notably, there was a significant reduction in the number of CD31-positive vessels present in tumors formed from U-251 cells re-expressing IC1, compared to the tumors from control U-251 cells (Fig. 5e).

### Inhibition of SK2 induces GBM cell death and reduces neoplastic growth in vitro and in vivo

**Fig. 6:**
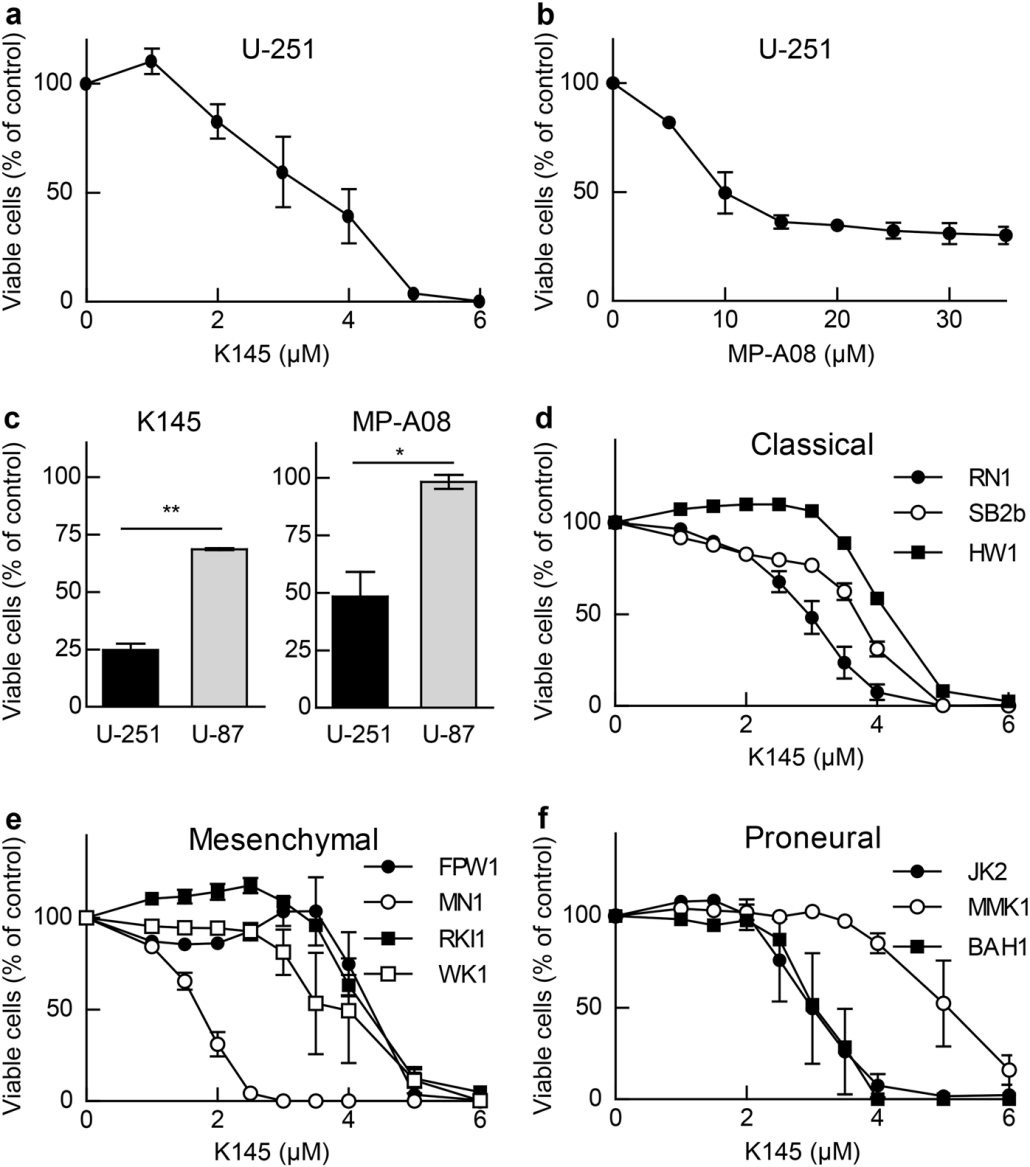
SK2 inhibition blocks growth of U-251 GBM cells and patient-derived cells from all molecular subtypes of GBM. Viability of U-251 GBM cells cultured for 72 h in the indicated concentrations of K145 (**a**) or MP-A08 (**b**) in the presence of 0.5% FBS was determined by MTS assay. Values are displayed as % vehicle control (DMSO), mean ± SEM (*n* = 3). **c** Comparison of the viability of U-251 and U-87 GBM cells cultured for 48 h in the presence of 6 μM K145 or 20 μM MP-A08 (*p < 0.05, **p < 0.01; Student's unpaired two-tailed *t*-test). Viability of primary GBM cells of low passage, established from patients with **d** classical, **e** mesenchymal and **f** proneural molecular subtype GBM, cultured for 72 h in the indicated concentrations of K145 in the presence of 0.5% FBS was determined by MTS assay. Values are displayed as % vehicle control (DMSO), mean ± SEM (*n* = 3–4)

Since our findings indicated that IC1 may act as a tumor suppressor in GBM via attenuating the plasma membrane localization and oncogenic signaling of SK2, we next examined the effects of SK2 inhibitors on GBM cells. Both K145, a selective SK2 inhibitor [20], and MP-A08, a dual SK1/SK2 inhibitor with greater potency against SK2 [21], effectively blocked the proliferation of U-251 cells (Fig. 6a, b). Notably, U-87 cells were less sensitive to both K145 and MP-A08 (Fig. 6c), consistent with the higher levels of IC1 and lack of plasma membrane-localized SK2 in these cells (Fig. 4). The effect of K145 (Fig. 6d–f) and MP-A08 (Supplementary Figure S5) was also assessed against a panel of low-passage primary patient GBM cell lines established from patients with classical, mesenchymal or proneural molecular subtypes of GBM. Indeed, consistent with the universal downregulation of IC1 across these three GBM subtypes (Supplementary Figure S2A), all GBM subtypes appeared similarly sensitive to SK2 inhibition.

**Fig. 7:**
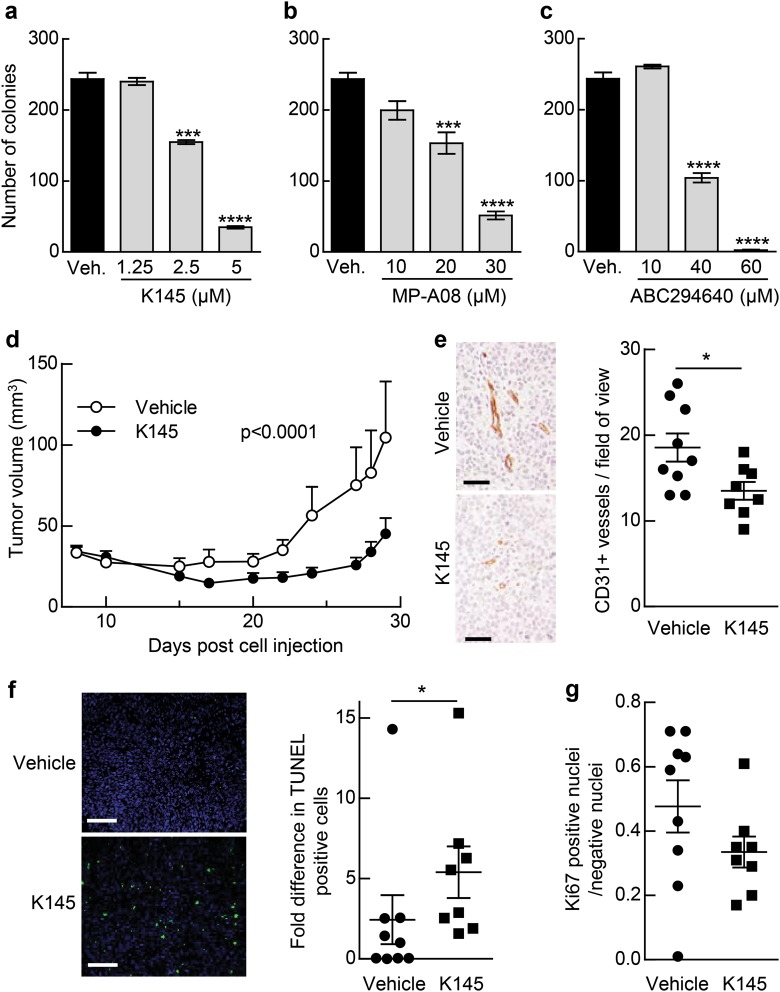
SK2 inhibition attenuates neoplastic growth of U-251 cells in vitro and in vivo. Anchorage-independent growth of U-251 cells was tested using colony formation assays in soft agar, in the presence of K145 (**a**), MP-A08 (**b**) and ABC294640 (**c**) or vehicle control (veh; 0.04% DMSO). Average colony numbers were quantified and are graphed as mean (±SD) from triplicate wells (***p < 0.001, ****p < 0.0001; Student's unpaired two-tailed *t*-test). **d** Mice bearing U-251 xenografts were administered 30 mg/kg body weight K145 (*n* = 8) or vehicle control (*n* = 9) intraperitoneally (i.p.) daily from day 8 following engraftment, and tumor volume assessed. Mean tumor volumes ± SEM are shown. Statistical analysis on all data points was performed by two-way ANOVA. After 3 weeks of treatment with K145 or vehicle control, tumors were excised and assessed for: **e** CD31-positive vessels by immunohistochemical staining, with an example of images for each group shown, and CD31-positive vessels per field of view quantified (*p < 0.05 by Student’s unpaired two-tailed t-test); **f** tumor cell apoptosis by fluorescent TUNEL and nuclear DAPI labeling and quantified by masking on DAPI-labeled nuclei and counting TUNEL-positive nuclei, with an example of images for each group shown (*p < 0.05 by two-tailed Mann–Whitney test for non-parametric distributions of data); and **g** tumor cell proliferation by Ki67 staining quantified by positive pixel analysis to yield the ratio of Ki67-positive vs. -negative nuclei for each sample (*p* = 0.066 by two-tailed Mann–Whitney test for non-parametric distributions of data). All scale bars = 100 μm

Notably, use of K145, MP-A08 and another SK2 inhibitor ABC294640 [22] in anchorage-independent colony formation assays resulted in a dose-dependent reduction in the number of colonies formed by U-251 cells (Fig. 7a–c), confirming that targeting SK2 has anti-neoplastic activity in these cells. We next examined in vivo efficacy of targeting SK2 on the growth of GBM xenografts in mice. NOD/SCID mice bearing U-251 flank xenografts were treated with K145 daily at 30 mg/kg for 3 weeks. K145 significantly reduced tumor growth (Fig. 7d) and, consistent with the effects of IC1 re-expression (Fig. 5e), targeting SK2 with K145 also reduced tumor vasculature as signified by a reduction in CD31-positive blood vessels (Fig. 7e). Furthermore, TUNEL (terminal deoxynucleotidyl transferase dUTP nick end labeling) fluorescence analysis and Ki67 immunohistochemistry revealed that K145 treatment resulted in both a significant increase in tumor cell apoptosis (Fig. 7f) and a trend towards reduced tumor cell proliferation (Fig. 7g), further consistent with the observed reduction in tumor growth.

## Discussion

**Fig. 8:**
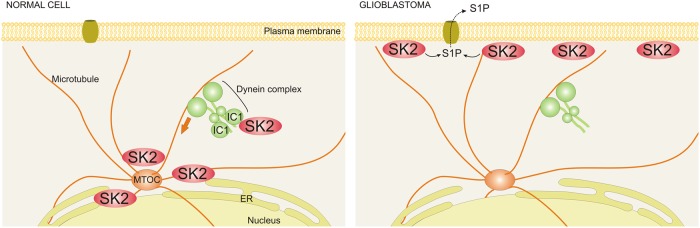
Schematic showing the mechanism by which dynein regulates the subcellular localization of SK2, and how the loss of IC1 in glioblastoma cells impacts on this. Dynein interacts with SK2 via IC1 to transport SK2 in a retrograde direction along microtubules towards the microtubule-organizing center (MTOC) located adjacent to the nucleus, resulting in reduced plasma membrane localization of SK2. Loss of IC1 expression commonly observed in GBM ablates the interaction of dynein with SK2, blocking its role in subcellular localization of SK2, resulting in increased plasma membrane localization of SK2 and release of S1P from the cell via S1P transporters

The roles and regulation of SK2 remain poorly understood. In this study we demonstrated that SK2 interacts with the cytoplasmic dynein complex, via dynein ICs, which transports SK2 away from the cell periphery and thus regulates its subcellular localization, which is critical for SK2 function (Fig. 8) [4]. Furthermore, we report that this novel SK2 regulatory mechanism has implications in GBM, where the expression of the IC1 subunit is heavily downregulated and correlates with poorer GBM patient survival. Indeed, our findings demonstrate an interplay between the downregulation of IC1 in GBM and an increase in SK2 plasma membrane localization, which was decreased by re-expression of IC1, resulting in reduced GBM tumor growth in vivo. Consistent with this, directly targeting SK2 with chemical inhibitors also showed anti-GBM activity in vitro, including in primary patient GBM cell lines representing the three molecular subtypes of GBM, and reduced GBM tumor growth in vivo. Overall, our work suggests that IC1 can act as a tumor suppressor in GBM via the regulation of SK2, and that SK2 is a promising target for examination as an anti-GBM therapy.

It is well accepted that the subcellular localization of the SKs, and hence the location of S1P production, can give rise to various opposing signaling outcomes [1, 4]. Therefore, the cellular functions of the SKs are largely dictated by their subcellular localization, which has previously been shown to be regulated by various interacting proteins. The calcium and integrin-binding protein CIB1 mediates the translocation of SK1 to the plasma membrane to promote survival and proliferative signaling [7]; however, it is unknown whether this mechanism also regulates SK2 plasma membrane localization. Translocation of the SKs to the plasma membrane has also been reported upon immunoglobulin E-mediated cross-linking of the FcεRI receptor on mast cells, mediated by an interaction with the Src family kinases Lyn and Fyn [23]. However, prior to this study, very little was known about the regulation of SK2 to regions of the cell where it is known to promote cell cycle arrest and cell death, such as the nucleus, endoplasmic reticulum and mitochondria [24–27]. Therefore, uncovering mechanisms by which SK2 is localized within the cell, particularly toward these organelles, is critical in understanding how the opposing roles of SK2 are regulated. As such, the studies outlined here have begun to decipher these mechanisms by identifying dynein as a critical regulator of SK2 subcellular localization which may therefore control many of the functions of this enzyme.

GBM is the most common and aggressive primary malignant form of brain cancer in adults, where patients have a median survival of less than 15 months after diagnosis and a 5-year survival rate of 3–5% [28]. The current standard of care for GBM patients is surgical resection of the tumor, followed by radiotherapy and administration of the chemotherapeutic agent temozolomide [28]. However, GBM remains incurable and there is clearly a need for more effective treatments. SK inhibitors have been proposed as promising therapeutic agents to use in combination with chemotherapy and/or radiotherapy in GBM [29], as SK-mediated conversion of pro-apoptotic ceramide, which is produced as a result of these therapies, to pro-proliferative S1P has been implicated in the resistance of GBM tumor cells to treatment-induced death [30, 31]. S1P levels are elevated in GBM tissue samples compared with normal gray matter [31], and S1P has been shown to mediate GBM cell proliferation and invasiveness through activation of S1P G protein-coupled receptors [32–34]. The relative importance in GBM of the two SK isoforms that produce S1P is, however, unclear. While SK1 is consistently upregulated in GBM and its expression correlates with poor patient survival [31, 35, 36], highly selective SK1 inhibitors have had surprisingly inconsistent effects on reducing GBM cell proliferation and viability [31, 37]. The efficacy of SK2-selective inhibitors has not been previously examined in GBM, potentially because the role of SK2 in cancer is generally less well understood and, unlike SK1, evidence of SK2 upregulation in GBM is inconsistent [31, 36, 38]. Interestingly, however, RNAi-mediated SK2 knockdown has been shown to reduce GBM cell proliferation and survival to a greater extent than SK1 knockdown [35]. Notably, SK2 is highly expressed in the brain [39]; in fact, SK2 expression in the brain is considerably higher than SK1 expression and, as such, SK2 is responsible for the majority of S1P production in this organ [40]. Here, we have demonstrated that SK2 plasma membrane localization and extracellular S1P production in GBM cells is negatively regulated by IC1, which is heavily downregulated in GBM. These findings provide an explanation for why SK2 contributes more to GBM cell proliferation and survival than SK1 [35], despite SK2 expression not being upregulated in GBM.

Strikingly, we have also demonstrated that targeting SK2 in GBM cells via re-expression of IC1, or by direct pharmacological SK2 inhibition, resulted in reduced tumor growth in vivo, possibly due, in part, to decreased tumor-associated angiogenesis. Drugs that target angiogenesis have emerged as promising anti-cancer therapeutics and, specifically, the antiangiogenic monoclonal vascular endothelial growth factor-blocking antibody, bevacizumab (Avastin), has been approved for use in patients with recurrent GBM. Given the important role of extracellular S1P in mediating tumor-associated angiogenesis [19], our findings suggesting an increase in plasma membrane-localized SK2 in GBM as a result of IC1 downregulation suggests that targeting SK2 in GBM may have beneficial antiangiogenic effects.

Another interesting concept to consider is whether the prominent plasma membrane localization of SK2 observed in GBM cells is seen universally in other cancer types where IC1 is significantly downregulated. According to our broad gene expression analysis, IC1 is significantly downregulated in many human cancers (Fig. 3a), notably in various subtypes of breast cancer across a number of datasets. SK2 has been previously shown to localize predominantly to the plasma membrane in MDA-MB-453 breast cancer cells, where it was found to be required for migration of these cells towards epidermal growth factor [26]. Furthermore, SK2 knockdown in MCF-7 breast cancer cells resulted in significantly reduced tumor growth in an in vivo xenograft model [41]. Therefore, it is tempting to speculate that the important interplay between SK2 and IC1 observed in GBM may also occur in other cancers, including breast cancer, where loss of IC1 is observed. However, given that IC1 is only highly expressed in the brain, heart and skeletal muscle [42], it will remain to be determined whether the ubiquitous IC2 isoform would compensate for the decrease in IC1 in other tissues.

Additional work is required to further understand and characterize the interaction between SK2 and dynein. Retrograde-directed movement of dynein results in its accumulation at the MTOC, and dynein is also found associated with organelles such as the golgi, lysosomes and endosomes [12]. Dynein, and the cargo that it transports, have been shown previously to exhibit peri-nuclear localization [43, 44] very similar to that demonstrated here by dynein IC and SK2 (Fig. 1d). Whether SK2 is being sequestered to specific peri-nuclear organelles, in either a house-keeping shuttling process or in response to certain stimuli, and how it is then transported away from these regions, are all questions that will require further attention. Indeed, in some situations this may represent a negative regulatory mechanism to prevent prolonged signaling of SK2 at the plasma membrane.

Overall, in this study we have identified a novel interaction between SK2 and the cytoplasmic dynein complex, and we have uncovered a new potential mechanism of SK2 dysregulation in cancer, whereby modulation of IC1 expression in GBM can regulate SK2 subcellular localization and ultimately affect tumorigenic potential. This work highlights the importance of investigating SK2 subcellular localization, and not simply whether it is upregulated, in determining whether pharmacologically targeting SK2 is likely to have therapeutic benefits in certain cancers. As such, this study offers a rationale for the use of SK2-specific inhibitors as potential anti-cancer therapeutics in GBM.

## Materials and methods

### Antibodies

The following primary antibodies were utilized: anti-FLAG (Clone M2 #F3165, Sigma-Aldrich), anti-HA (#H3663, Sigma-Aldrich), anti-DYNC1IC (clone 74.1, #MAB1618, Millipore), anti-dynactin p150 (#SC-135890, Santa Cruz Biotechnology), anti-α-tubulin (#ab7291, Abcam), rabbit anti-DYNC1I1 (#13808-1-AP, Proteintech), anti-DYNC1I2 (#ab96288, Abcam), anti-DYNC1LIC1 (#ab157468, Abcam), anti-SK2 (#SP4621, ECM Biosciences; and #17096-1-AP, Proteintech), anti-GFP (#600-101-215, Rockland Immunochemicals), anti-PECAM-1 (CD31; #SC-1506, Santa Cruz Biotechnology) and anti-Ki67 (#VP-K452, Vector Laboratories).

### Cell culture

HEK293 cells were purchased from ATCC (Manassas, VA, USA), and cultured as previously described [45]. GBM cells were cultured in minimal essential media (MEM; Life Technologies), containing 10% fetal bovine serum (FBS), 2 mM l-glutamine, 1 mM sodium pyruvate, 1% non-essential amino acids, penicillin (1.2 mg/ml) and streptomycin (1.6 mg/ml). Patient-derived primary GBM cell lines were established as previously reported [46], and cultured in serum-free KnockOut Dulbecco's modified Eagle's medium/F-12 basal medium supplemented with StemPro NSC SFM supplement, 2 mM GlutaMAX-I, 20 ng/ml epidermal growth factor, 20 ng/ml fibroblast growth factor-β (Life Technologies) on flasks coated with 1% Matrigel Matrix (Corning). All cells were grown at 37 °C with 5% CO_2_ in a humidified incubator and periodically tested for mycoplasma contamination.

### Mammalian expression constructs

Mammalian expression construct encoding SK2 was generated previously [47]. Human SK2b (Genbank Accession number NM_020126) was PCR amplified from placenta cDNA and FLAG epitope-tagged at the 3′ end by PCR with oligonucleotide primers 5′-TAGGATCCGCCA-CCATGAATGGACACCTTGAAGCA-3′ and 5′-TAGAATTCACTTGTCATCGTCGTCCTTG-TAGTCGGGCTCCCGCCCCGGGCA-3′. The resultant PCR product was cloned into pcDNA3 (Invitrogen) following digestion with *Bam*HI and *Eco*RI. SK2b was PCR amplified with primers 5′-TAGAATTCATGAATGGACACCTTGAAGC-3′ and 5′-TAGAATTCAGGGCTCCCGCC-CCG-3′, digested with *Eco*RI and cloned into pGBKT7 (Clontech).

Human DYNC1I1 (NM_001135556) and DYNC1I2 (NM_001271786) were amplified from placenta cDNA and hemagglutinin (HA) epitope-tagged at the 3′ end by PCR with oligonucleotide primers 5′-TAGGATCCGCCACCATGTCTGACAAAAGTGACTTA-3′, 5′-TAGAATTCAAGCGTAATCTGGAACATCGTATGGGTAGGCAGATAACTCAACAGTGC-3′ and 5′-TAGGATCCGCCACCATGTCAGACAAAAGTGAATTAAAG-3′, 5′-TAGAATTC-TCAAGCGTAATCTGGAACATCGTATGGGTAAGCAGGTATTCGGGTAGCTG-3′ respectively. PCR products were cloned into pcDNA3 following digestion with *Bam*HI and *Eco*RI.

pCX^NEO^-IRES EGFP [8] was digested with *Xho*I and *Sac*I, blunted and re-ligated to produce pCX1^NEO^. A modified polylinker was introduced following digestion with *Eco*RI and ligation of annealed kinased oligonucleotides 5′-AATTCGGTACCGAGCTCGCTAGCGCGGCCGCCTC-GAGC-3′ and 5′-AATTGCTCGAGGCGGCCGCGCTAGCGAGCTCGGTACCG-3′ to produce pCX3^NEO^. EGFP was PCR amplified with oligonucleotide primers 5′-TAGAATTCATGGTGA-GCAAGGGCGAG-3′ and 5′-TACTCGAGCGGCCGCTCACTTGTACAGCTCGTCCATGC-3′. The link/EGFP product was digested with *Eco*RI and *Not*I and cloned into pCX3^NEO^. DYNC1I1 was PCR amplified with T7 and 5′-TAGAATTCGGGGGCAGATAACTCAACAGTGC-3′ and cloned into pCX3^NEO^-link/EGFP following digestion with *Bam*HI and *Eco*RI to produce pCX3^NEO^-DYNC1I1-EGFP. Sequencing verified integrity and orientation of all cDNAs.

### Yeast two-hybrid screen

The yeast two-hybrid screen, using full-length human SK2b as bait, was performed using the Matchmaker Gold Gal4 Two-Hybrid System (Clontech) and a universal human normalized cDNA library (Clontech) according to the manufacturer’s instructions, and as previously described [48].

### Transfection, immunoblotting and immunoprecipitation

Transient transfection of DNA, or co-transfection of DNA and siRNA into cells, was performed using Lipofectamine™ 2000 (Invitrogen), as per the manufacturer’s protocol. Cell lysate preparation, immunoblotting and immunoprecipitation was performed as previously described [9, 45]. For immunoprecipitation of endogenous SK2, C57/Bl6 mouse brain lysates were made as previously described [49], and immunoprecipitation was performed using anti-SK2 antibodies (ECM Biosciences).

### siRNA knockdown

RNAi-mediated DYNC1I1 and DYNC1I2 knockdown was performed using methods previously described [45], using ON-TARGETplus non-targeting siRNA pool or human DYNC1I1 and DYNC1I2 ON-TARGETplus SMARTpool siRNA (Dharmacon) targeting the following sequences for DYNC1I1: GGAAGGCACUGUUGAGUUA, GGAAAUUCGUGCUAACAGA, CAAGGGAAGUAGUGUCCUA, CGGGAGACGUCAAUAACUU, and for DYNC1I2: GUAAAGCUUUGGACAACUA, GAUGUUAUGUGGUCACCUA, GCAUUUCUGUGGAGGGUAA, GUGGUUAGUUGUUUGGAUU.

### Generation of stable cell lines

To generate stable cell lines expressing DYNC1I1 as a GFP-fusion protein, U-251 cells were transfected with pCX3^NEO^-DYNC1I1-EGFP, or empty vector. At 48 h after transfection, cells were sorted for low-level GFP-positive cells using a FACSAria II cell sorter (BD Biosciences). Stable GFP-positive pooled cell populations were obtained by sorting for GFP another two times.

### Quantitative reverse transcriptase-polymerase chain reaction (qRT-PCR)

RNA extracted from GBM cells was used to generate cDNA using the QuantiTect reverse transcription kit (QIAGEN) according to the manufacturer’s protocol. Gene expression was assessed by qRT-PCR using the QuantiTect SYBR Green PCR kit (QIAGEN) as previously described [49], using human *DYNC1I1* primers TAAAGTTGGCCAGGACTCAG and CCAGGGCTCTTTC-AATTACC, or human *DYNC1I2* primers CCAGTTATGGCTCAACCCAA and TCAGAGTGCAAGATTTGTTGC. Relative gene expression was normalized to GAPDH gene expression and analyzed by the comparative quantitation method as previously described [49].

### Immunofluorescence

Immunofluorescence staining and confocal imaging was performed as previously described [45]. Co-staining of F-actin was performed by incubating cells with AlexaFluor 647 Phalloidin (Molecular Probes) for 30 min at room temperature. TUNEL analysis on tumor tissue was performed as previously described [21].

### Immunohistochemistry

PECAM-1(CD31) expression in formalin-fixed paraffin-embedded tumor tissues was assessed as previously described [9]. Color development was carried out using 3,3′-diaminobenzidine tetrahydrochloride (DAB) reagent (Dako). For Ki67 imaging bright field scanning was performed using a NanoZoomer slide scanner (Hamamatsu). Images were processed using ImageJ (National Institutes of Health) or Imagescope (Aperio) software in a blinded manner as previously described [50, 51].

### Duolink® protein interaction assay

Interactions between endogenous SK2 and dynein IC in HEK293 cells were assessed with the Duolink® in situ proximity ligation assay kit (Sigma-Aldrich), as previously described [49].

### S1P formation assay

The rate of extracellular S1P formation from intact cells was determined as previously described [52]. Briefly, cells were seeded into 6-well plates and were grown in MEM containing 10% FBS overnight. Cells, at 80% confluence, were then labeled with 0.5 µCi of [^3^H]-sphingosine (Perkin-Elmer, Rowville, VIC, Australia), delivered in serum-free media (7.5% final serum concentration on cells), for 30 min. The conditioned media were then collected and extracellular [^3^H]-S1P generated in the conditioned medium was extracted via a modified Bligh–Dyer extraction [52], and analyzed by scintillation counting. Analyses were performed in quadruplicate with data normalized to cell number.

### Cell viability assays

Relative viable GBM cell numbers after SK2 inhibitor treatment were determined by CellTiter MTS assay (Promega) as per the manufacturer’s protocol.

### Colony formation

Colony formation assays in soft agar were performed as previously described [9]. After 14–21 days, images were taken of five random fields of view per well using an Olympus MVX10 microscope. Average colony number and size was quantified using ImageJ software.

### In vivo tumor models

Experiments involving mice were approved by the SA Pathology/CALHN Animal Ethics Committee. To examine the effect of IC1 re-expression on tumor growth, 5 × 10^6^ U-251 cells were injected subcutaneously into the flank of 9-week-old female NOD/SCID mice. Mice were examined daily and caliper measurements of palpable tumors were taken. To examine the effects of K145 on tumor growth, U-251 flank xenografts were established in 8-week-old female NOD/SCID mice as described above, and tumors measured daily. At 8 days post cell injection, mice were randomized into two groups and administered K145 or 40% polyethylene glycol 400 vehicle control intraperitoneally daily. At the endpoint of both models, mice were humanely killed, tumors excised, fixed in 10% formalin and paraffin embedded or used for lysate preparation.

## Electronic supplementary material

Supplementary Figures
