# Supplementary material for: Cytoplasmic dynein regulates the subcellular localization of sphingosine kinase 2 to elicit tumor-suppressive functions in glioblastoma
Source: Oncogene. 2018 Sep 24;38(8):1151–65. doi: 10.1038/s41388-018-0504-9 (PMC6363647; doi:10.1038/s41388-018-0504-9)
Supplement: Supplementary file 1 — Supplementary Figures [file 41388_2018_504_MOESM1_ESM.pdf]

Supplementary Material

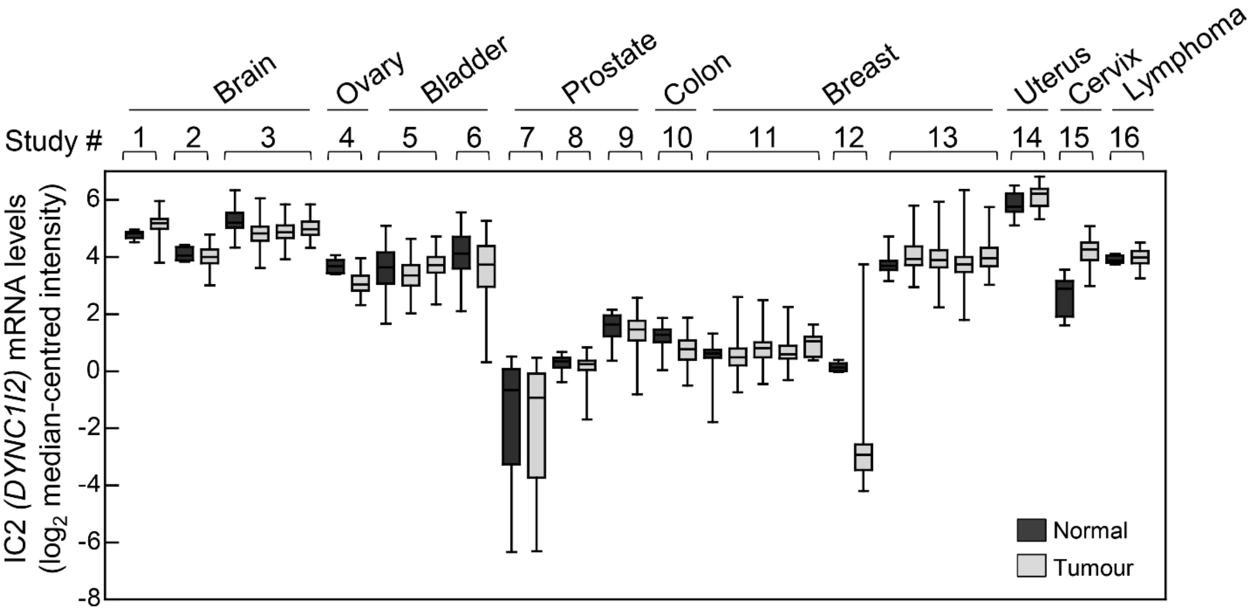

Supplementary Figure S1: IC2 expression does not vary considerably in human cancer patient data sets where IC1 is downregulated

Box plots showing IC2 (*DYNC1I2*) mRNA levels in various human cancer patient datasets where significant downregulation of IC1 (*DYNC1I1*) was observed (Figure 3). Data were extracted from the Oncomine database (details of studies used are described in Figure 3).

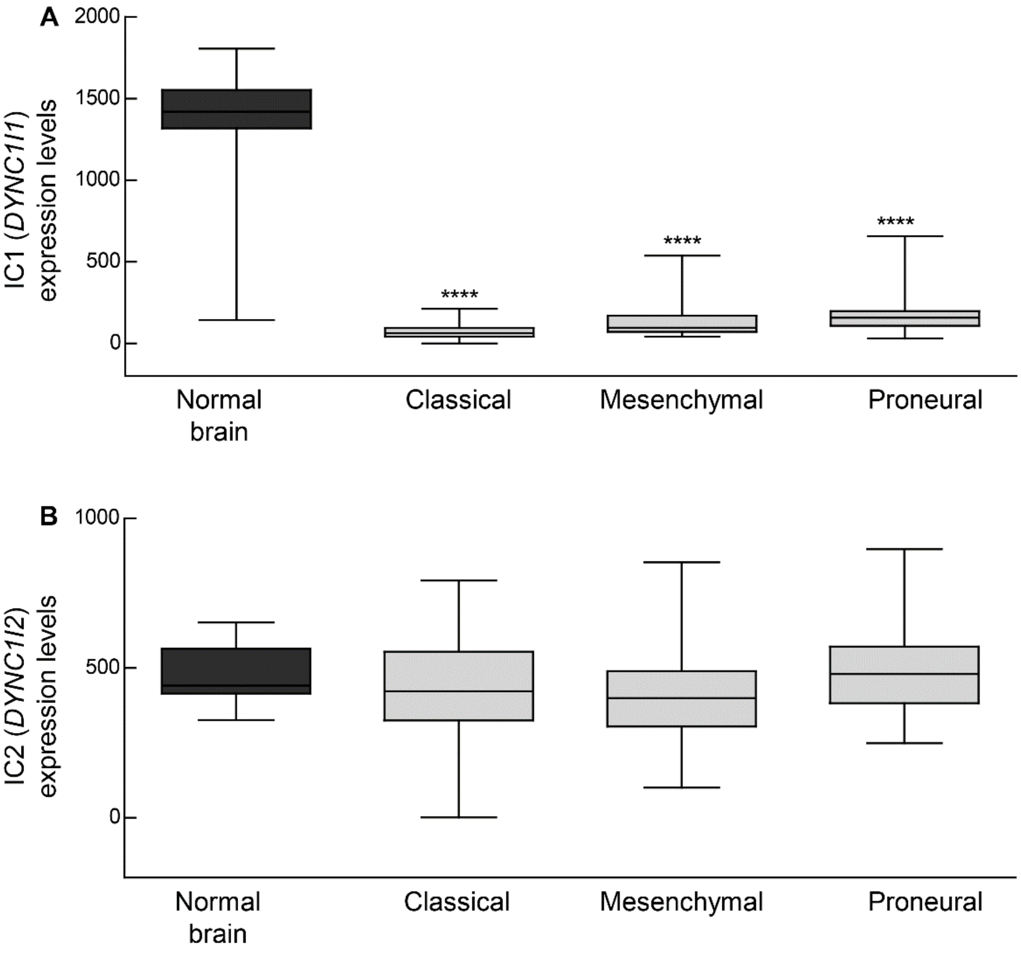

Supplementary Figure S2: IC1 is significantly downregulated in all GBM subtypes, whereas IC2 expression is unchanged

Box plots showing (A) IC1 (*DYNC1I1*) and (B) IC2 (*DYNC1I2*) mRNA levels in human patient samples from the four GBM subtypes: Classical, Mesenchymal and Proneural, compared to normal brain tissue. Data were extracted from the TCGA Glioblastoma data set, from the expression box plot (Affymetrix Human Exon 1.0 ST) platform available from the Project Betastasis database (**** $p<0.0001$; Student's unpaired two-tailed t-test). Data present in the Project Betastasis database from the previously designated Neural GBM subtype has been omitted since this GBM subtype is no longer recognised.[16]

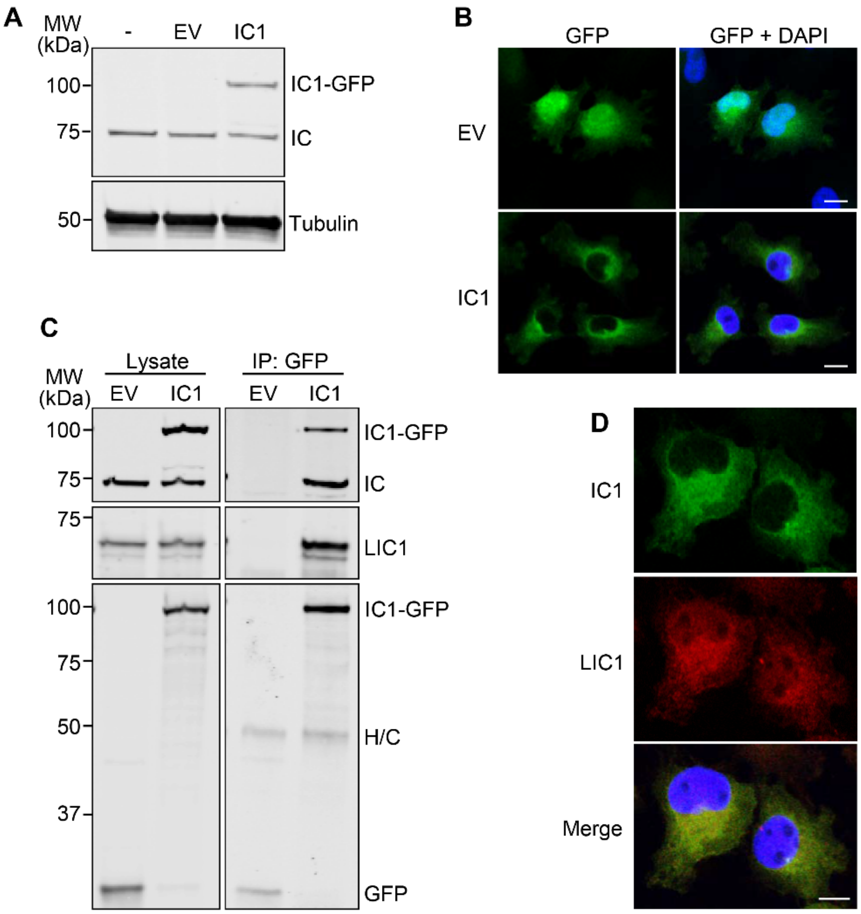

Supplementary Figure S3: Low-level exogenous IC1-GFP incorporates into endogenous dynein complexes

(A) Lysates from parental U-251 cells, or U-251 stable cell lines expressing GFP alone (empty vector; EV) or IC1-GFP, were subjected to immunoblot analyses with antibodies against dynein IC and α-tubulin. Blots shown are representative of three independent experiments. (B) U-251 cells stably expressing GFP (EV) or IC1-GFP were imaged using confocal microscopy, and exogenous proteins were visualized via their GFP tag (green). Nuclei were stained with DAPI (blue). Images are representative of more than 100 cells, from three independent experiments. Scale bar = 10 μm. (C) Lysates were prepared from U-251 cells stably expressing GFP alone (EV) or IC1-GFP, and these exogenous proteins were then immunoprecipitated using anti-GFP antibodies. Co-immunoprecipitated endogenous dynein intermediate chains (IC) and light intermediate chain 1 (LIC1) were detected by immunoblotting with anti-IC and anti-LIC1 antibodies, respectively. Expression levels of these proteins in the lysates were also confirmed by immunoblotting with their respective antibodies (*Lysate*). Lysates and immunoprecipitates were probed with anti-GFP antibodies to confirm expression and pull-down of IC1-GFP or GFP. Blots shown are representative of three independent experiments. H/C designates the heavy chain IgG band. (D) Co-localization of IC1-GFP and endogenous dynein light intermediate chain 1 (LIC1) in the U-251 stable cell line was demonstrated by confocal microscopy. IC1-GFP was visualized via its GFP fusion (green) and LIC1 via immunofluorescence staining of the endogenous protein using anti-LIC1 antibodies (red; 1:250). Nuclei were stained with DAPI (blue). Images are representative of more than 100 cells, from three independent experiments. Scale bar = 10 μm.

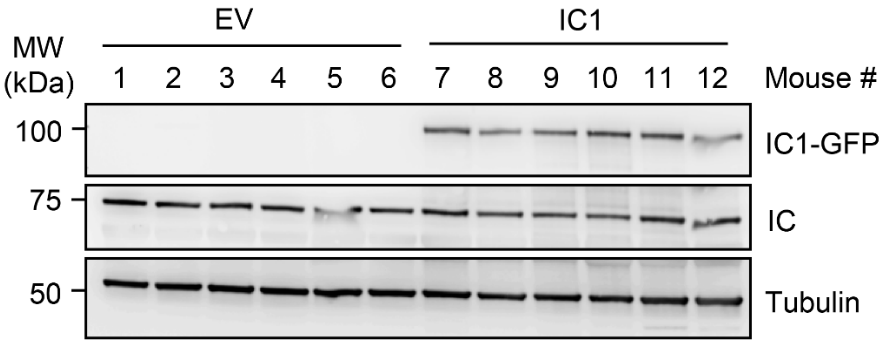

Supplementary Figure S4: Ectopic IC1 expression was observed in all tumors arising from cells re-expressing IC1

Lysates from homogenized tumor tissue obtained from the experiments detailed in Figure 5d were subjected to immunoblot analyses with antibodies against GFP, dynein intermediate chains (IC) and α-tubulin. Each lane represents a single tumor sample from one mouse.

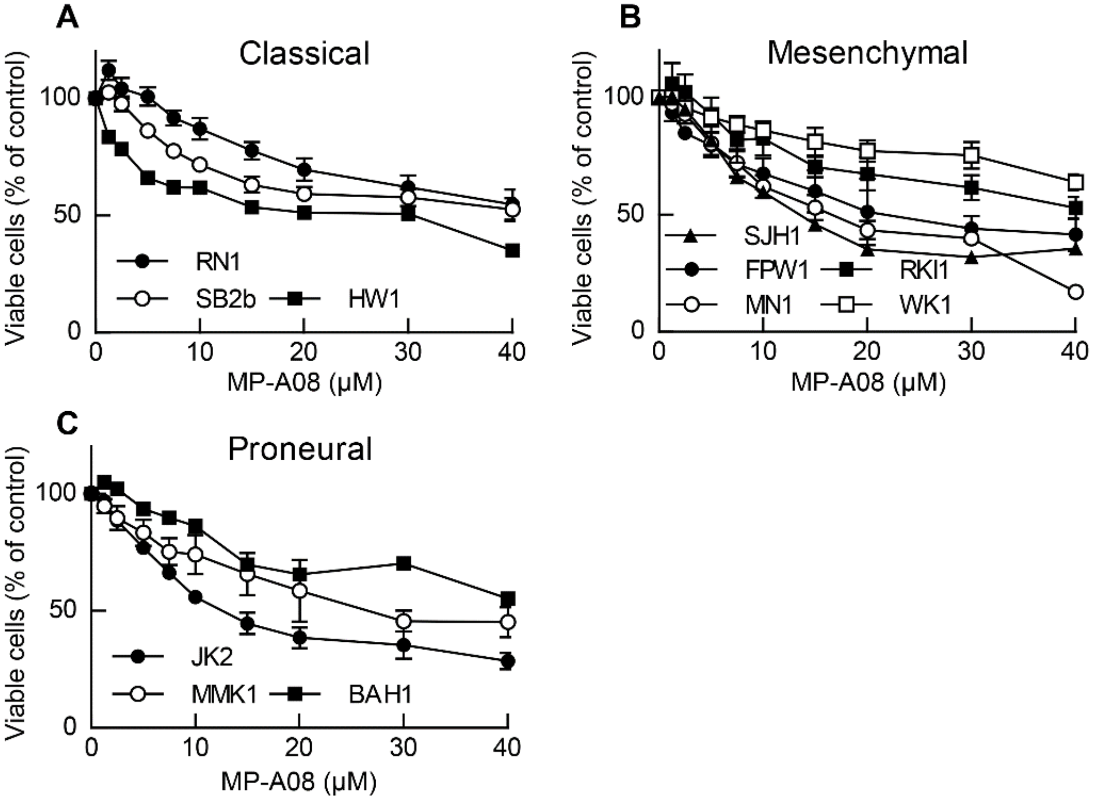

Supplementary Figure S5: SK2 inhibition blocks growth of patient-derived cells from all four molecular subtypes of GBM

Viability of primary GBM cells of low-passage, established from patients with (A) Classical, (B) Mesenchymal, and (C) Proneural molecular subtype GBM were determined by MTS assay with varying concentrations of MP-A08 for 72 h. Values are displayed as % vehicle control (DMSO), mean ± SEM (n=3–4).
